# Could minor neurological dysfunction be a type of non-cerebral palsy motor impairment?

**DOI:** 10.1055/s-0045-1809359

**Published:** 2025-06-01

**Authors:** Ana Paula Hamad, Carla Andrea Tanuri Cardoso Caldas, Isabella Cristina Mendes de Sá e Silva, Marina Estima Neiva Nunes

**Affiliations:** 1 Universidade de São Paulo, Faculdade de Medicina de Ribeirão Preto, Departamento de Neurociências e Ciências do Comportamento, Ribeirão Preto SP, Brazil.

**Keywords:** Neurodevelopmental Disorders, Infant, Premature, Motor Skills Disorders, Psychomotor Disorders, Neurological Examination, Cognitive Dysfunction

## Abstract

Non-cerebral palsy motor impairments have been increasing over time, especially in preterm-born children, with a variety of labels, including, but not limited to, developmental coordination disorder and minor neurological dysfunction. They may not only negatively affect the child in their daily life activities, but are also associated with cognitive, emotional, and behavioral disorders, as well as decreased academic performance.﻿ Since there is a paucity of literature on non-cerebral palsy motor impairment, the main objective of the present study is to assess minor neurological dysfunction in the context of neurodevelopment. We reviewed the medical literature using the following search terms: *preterm infants* ; *follow-up* ; *non-cerebral palsy motor impairment or disability* ; *minor neurological dysfunction* ; *developmental coordination disorder* ; *neurological soft signs* ; and *Touwen Neurological Examination* . We compiled evidence-based data on minor neurological dysfunction critical domains, diagnosis, etiological factors and the influence of age and puberty in minor neurological signs. We also listed the cohort studies that used the Touwen Neurological Examination for premature or term-born follow-up, including those that suggest an association between minor neurological dysfunction and developmental coordination disorder as a motor impairment beyond cerebral palsy. Moreover, we found significant evidence of a correlation involving minor neurological dysfunction and cognitive, behavioral, and learning abilities, which, all in all, means that these children particularly deserve special attention regarding social, educational and healthcare resources. Furthermore, since motor activity seems to play an important role in the physical, mental, and neurological health of typical children, different approaches to education could have a positive impact on neurological development.

## INTRODUCTION

Longitudinal studies have suggested that, rather than an increase in the rates of cerebral palsy (CP), the prevalence of non-CP motor impairments has been increasing over time. 1 2 3 4 5 Motor impairments beyond CP were not as well known until there was a unification of concepts, such as clumsiness, developmental dyspraxia, and perceptual-motor dysfunction, for developmental coordination disorder (DCD). 6 Since DCD shares most of CP's risk factors, some authors call it *non-CP motor impairment* . The definition of DCD was endorsed in the International Consensus Meeting in the city of London, Ontario, Canada, in 1994, as a neurodevelopmental disorder characterized by the acquisition and execution of coordinated motor skills substantially below what is expected, given the individual's chronological age and opportunity for skill learning and use; and manifested by clumsiness, slowness, or inaccuracy of performance of motor skills that interfere with daily life and impact academic, occupational, recreational or social activities. 6 7 Afterwards, this term was incorporated by the International Classification of Diseases (ICD), the *Diagnostic and Statistical Manual of Mental Disorders* (DSM), and the International Classification of Functioning, Disability and Health (ICF), as well as by guidelines, such as those of the European Academy of Childhood Disability (EACD). 6 7 With the unification of terms, much has been studied about DCD and its neurobiology, epidemiology, diagnosis, clinical implications, and treatment. However, regarding neurological semiology in the era of technological advances, it is necessary to recover established concepts.

The traditional pediatric neurological examination is normal in almost all cases of neurodevelopmental disorders, or it yields isolated findings devoid of practical significance. Several child neurological assessment tools have been developed to access children by specific age. Few are intended to capture subtle neurological signs, which may be present in neurodevelopmental disorders, and represent an underlying brain pathology. 8 9

One of them is the Touwen Neurological Examination, which was developed to identify a set of subtle neurological signs that can be categorized and define minor neurological dysfunction (MND). 9 According to Hadders-Algra 10 11 and Hadders-Algra et al. 12 , who studied the subject the most, the occurrence of MND in early childhood may be associated with motor (DCD), cognitive, and behavioral impairments.

Minor neurological dysfunction, especially in its complex manifestation (complex MND), may represent a brain pathology involving the cortico-striato-thalamo-cortical and cerebello-thalamo-cortical pathways, and it shares the same risk factors for CP and DCD. 10 11 In particular, the occurrence of complex MND in children with DCD reaches almost 80%, 13 and the occurrence of DCD in children with complex MND reaches nearly 100% 14 when it comes to premature children. Thus, MND could be particularly related to the diagnosis of DCD, or non-CP motor impairment, as a motor impairment beyond CP.

Since these are our beliefs, we decided to review the subject to compile information for child neurologists and other professionals, either interested in assistance or for research purposes. Additionally, it is our opinion that this knowledge has implications for preventive healthcare and education policies. There is a paucity in the literature of papers on non-CP motor impairment. The articles also differ in terms of nomenclature and approaches. There are even fewer articles on MND.

For the current narrative review, and with a focus on our main objective (to contextualize MND), we reviewed the international medical literature using single and/or combined keywords, as follows: *preterm infants* ; *follow-up* ; *non-CP motor impairment* ; *non-CP neuromotor disabilities* ; *minor neurological dysfunction* ; *developmental coordination disorder* ; *neurological soft signs* ; and *Touwen Neurological Examination* . Articles in English published since 1970 were accessed through the main available databases (SciELO, Web of Science, Scopus, PubMed, and Embase).

We selected the articles with the greatest relevance to the proposed topic, based on qualitative criteria, to better understand the subtle neurological findings and MND; to characterize the Touwen Neurological Examination and MND features as follow-up resources in populational studies; to classify MND and DCD as non-CP motor impairments; and to examine the association of MND with learning, behavioral and cognitive impairments, and its possible implications for preventive healthcare and education policy. Articles including CP, intellectual disability, autism spectrum disorder, and toddler assessments were excluded.

## SUBTLE NEUROLOGICAL FINDINGS AND MND

Subtle neurological findings have also been reported in the literature 10 15 as *neurological soft signs* or *minor disabilities* . Subtle neurological signs represent findings relating to tone, posture and reflexes, which neither constitute the spasticity of CP at one extreme nor hypotonia of a neuromuscular disease at the other. Furthermore, they are represented by overflow movements (movements not involved with the main task, which “overflow” from the contralateral side, such as mirror movements), involuntary movements (such as tremor and choreiform movements of the extremities), and dysmetria (difficulty in correcting the trajectory of an intentional movement with precision involving the coordination of extremities), among others. 15

Initiatives towards standardized and age-adequate neurological examination techniques focusing on mild motor dysfunctions in infants without CP emerged in the 1970s and are still used today. In these cases, the examination technique must consider the natural history of the neurological repertoire development that occurs throughout childhood. 10 16 These focused neurological examinations can either detect functional defects or substantiate their absence, as well as discriminate deviations that stem from a lag in maturation speed. 16 The main ones are the Examination of the Child with Minor Neurological Dysfunction, also named the *Touwen Neurological Examination* , 9 the Hempel Examination, 17 18 the Physical and Neurological Examination for Soft Signs (PANESS), 19 and the revised Neurological Examination for Subtle Signs (NESS). 20

The Hempel examination assesses MND in children from 18 months up to 4 years. It may be considered the preschool-age equivalent of the Touwen Neurological Examination, which is the age-specific tool to assess MND in children from 4 years onwards. 9 18

In isolation, subtle neurological findings may have no meaning and are not valued in the traditional pediatric neurological examination. However, according to Touwen, 9 a set of neurological soft signs composes a motor domain or cluster, and a set of clusters can lead to what is conventionally named *minor neurological dysfunction* . The classification, associations, and implications of MND will be discussed in the next section.

### Minor neurological dysfunction

Especially for school-age children, the tests published by Touwen 9 are some of the most widely used in longitudinal studies. Despite the intertwin part of the traditional neurologic examination, due to its length (15–20 minutes), it is less applied in the daily practice. However, it remains a valuable resource in clinical research settings. 12 16 21 22 23 24 25 26 27

The complete evaluation includes 58 items distributed among 10 subsystems: sensorimotor apparatus, posture, balance of trunk, coordination of the extremities, fine manipulative ability, dyskinesia, gross motor functions, quality of motility, associated movements, and visual systems. 23 In the final profile, these 10 subsystems can be grouped into 6 clusters: posture and muscle tone, reflexes, choreiform dyskinesia, coordination and balance, fine manipulative ability, and miscellaneous dysfunctions. Such clusters are classified by each functional subsystem of the nervous system implemented in the clinical practice, to highlight MND in children 16 23 ( Table 1 ).

**Table 1:** Functional (neurobehavioral) clusters of MND, based on the Touwen Neurological Examination 9 (1979) for children aged ≥ 4 years and older

Cluster of dysfunctions	Based on	Criteria for dysfunctional cluster
Dysfunctional muscle tone regulation	Muscle tone and posture during sitting, crawling, standing, and walking	One or more of the following: – consistent mild deviations in muscle tone; – consistent mild deviations in posture
Reflex abnormalities	Intensity and/or threshold or asymmetry in: – biceps reflex; – knee jerk; – ankle jerk. Footsole response: uni- or bilateral Babinski sign	Presence of at least two signs
Choreiform dyskinesia	Spontaneous motor behavior; test with extended arms; movements of face, eyes, tongue	Presence of at least one of the following – marked choreiform movements of distal and facial muscles; – slight or marked choreiform movements of proximal muscles, eyes or tongue
Coordination problems	Finger-nose test; fingertip-touching test; diadochokinesis; kicking; knee-heel test; reaction to push (sitting, standing); Romberg; tandem gait; standing on one leg	Presence of age-inadequate performance on at least two tests
Fine manipulative ability	Finger-opposition test: – smoothness; – transition. Follow-a-finger test; circle test;	Presence of age-inadequate performance on at least two tests
Rarely-occurring miscellaneous disorders	motor behavior of face, eyes, pharynx, and tongue; associated movements during diadochokinesis, finger-opposition test, walking on toes or heels	Evidence of at least one of the following: – mild cranial nerve palsy; – excessive amount of associated movements for age

Note: Adapted from Hadders-Algra et al. 34

By the end of the Touwen examination, the presence of MND and its degree of severity can be ascertained if at least one of the defined clusters is altered. It is important to emphasize that the presence of a single sign of dysfunction does not define MND. This means that the presence of a cluster of signs of dysfunctions is essential to the diagnosis of MND. 14 22 “This is one of the reasons why minor signs only have clinical significance if they co-occur with functionally-related signs, i.e. if they occur clusterwise” 22 ( Table 1 ).

Minor neurological dysfunction can also be classified as simple or complex. In addition, the criteria for one or the other are age-specific. Before the onset of puberty, the distinction is based on the number of dysfunctions: a child is neurologically normal if all the clusters are normal; they present simple MND if one or two clusters are deviant; and they present complex MND if more than two clusters are scored as abnormal. After the onset of puberty, when most children with MND present with only a single cluster, the discrimination is based on the type of dysfunction present– it becomes a qualitative evaluation, either as postpuberal hypotonia or choreiform dyskinesia (as a sign of simple MND) or as fine manipulation or coordination issues (as a sign of complex MND). 10 11 12 14

Overall, simple MND may be regarded as the expression of a normal but non-optimally functioning nervous system (minor neurological difference). The fact that it seems to be associated with a moderately-increased risk of developing learning and behavioral problems corroborates this hypothesis; in contrast, complex MND can be associated with the presence of substantial neural dysfunction which induces vulnerability to neurodevelopmental disorders. 10 11 14

Regarding the etiology and risk factors, simple MND might be largely epigenetically-determined, as its genesis appears to vary from genetic constitution to acquired lesions due to stress in early life, with disturbances in the monoaminergic system. Few perinatal risk factors have been associated with the development of simple MND, such as preterm birth, mild to moderate perinatal asphyxia, and severe intrauterine growth retardation. Frequent colds, family history of neuropsychiatric disorders, and the male gender have also been associated. Differently from simple MND, complex MND has stronger pre- and perinatal roots, as it is mainly associated with neonatal neurological deviancy, birth before 33 weeks of gestation, and a low obstetrical optimality score. Complex MND appears to be the result of minor brain lesions at an early age, similar to CP. The male gender has also been highlighted as a risk factor for complex MND, which sounds coherent, since it is well reported that boys are more vulnerable to neonatal insults than girls. Finally, socioeconomic status seems to also play an important role in the association between neonatal neurological morbidity and complex MND. 10 11 14 The association with these perinatal risk factors is a strong reason for MND, as DCD, to be recognized as non-CP motor impairment.

In terms of the proposed pathophysiology, the literature suggests that the front-striatal dopaminergic system may operate in a non-optimal manner. Early-onset stress may induce permanent functional changes in the monoaminergic systems in the basal ganglia, cerebellum, hippocampus, and frontal cortex. 10 11 14 Since the cerebellum and periventricular structures, such as the caudate nucleus, exhibit developmental activity in the last trimester of pregnancy, they become more vulnerable to harmful conditions. Besides, during this period, the central white matter is known to be a major site for lesions. Some part of complex MND's origin might be due to an interruption of connecting fiber systems, such as the corpus callosum or the descending systems in the internal capsule. Interestingly, the type of dysfunctions that play a prominent role in complex MND (fine manipulative ability, and coordination) may reflect dysfunction of the cortico-striato-thalamo-cortical and cerebello-thalamo-cortical pathways. These circuitries appear to be implicated in sensorimotor aspects of motor programming, movement planning, program selection, motor memory and cognitive tasks involved in learning, and attention. This reinforces the strongest association of complex MND with cognitive, learning and attentional difficulties. 10 11 14

### MND and population studies

To better elucidate these concepts, Hadders-Algra has been substantially contributing to the knowledge of MND. 28 29 Throughout several papers and books, she and other collaborators managed to emphasize the importance of MND's diagnosis, as well as its critical domains, as they elaborate on etiological factors, the role of age and puberty in the minor neurological signs, and the impact of the disorder on cognitive, behavioral and learning abilities. 10 11 12 29 30 31 32 33 34 35 36 37 38 39

Hadders-Algra and some collaborators, in a pioneering way, studied the MND features from the data of the Groningen Perinatal Project (GPP), a long-term follow-up project of a 3-year birth cohort (from 1975–1978) of 3.162 infants born at the University Hospital in Groningen, Netherlands. 29 31 Follow-up examinations using the Touwen's standardized and age-adequate neurological examination were performed at the ages of 1.6, 4, 6, 9, 12, and 14 years, with emphasis on identifying MND. 10

The results of the GPP underline that MND is age-dependent due to the increase in the complexity of neural circuitries and to the developmental changes in the nervous system. The latter not only influences the normal neurological repertoire, which in turn enables the emergence of increasingly-complex motor and cognitive functions, but also affects the presentation of the dysfunctions, which can only be detected in those functions to which the brain development provides functional access. 10

The prevalence of MND in toddlers is low. A major finding reported 10 during the preschool period was a high recovery rate in infants with abnormalities at the neonatal stage: while 10% developed CP, 12% showed MND at the age of 4 years. Nevertheless, these rates were significantly higher than those found in children neurologically normal at birth (0% with CP and 7% MND). 10 Since clinical features change throughout the child's development until puberty, MND may not be present in young children, in which case it may result in worse outcomes in terms of cognition, behavior, and learning. 10 11 However, knowledge about the significance of the signs of MND during the toddler period is still scarce. 11 12 14 The condition may be present in typical toddlers and preschool children, and it is believed that its main pathological significance is its persistence into school age and adolescence, when in fact it could be considered a marker of atypical neurodevelopment. However, as brain structure and connectivity are in the process of formation and maturation throughout childhood, the identification of MND at preschool age could already indicate the need for greater investment in motor enrichment opportunities. 15

Throughout the childhood, the frequency of MND showed a steady increase: 20% of normal newborn infants, 25% of infants with mild abnormality, and 35% of neonates with severe abnormality showed MND at 9 years of age, reaching its peak shortly before the onset of puberty. 10

Notwithstanding, puberty appears to induce a substantial decline in the rate of MND; by the age of 14, only 7 to 8% of children exhibited MND, most of them presenting with only one dysfunctional cluster. The GPP team 10 29 proposed that MND decline around puberty might be mediated by age-related hormonal changes, as the rise in gonadal hormones can improve motor performance, as well as play a positive role in brain injury by inducing axonal sprouting and enhancing synaptic transmission. Likewise, thyroxine, which increases during puberty, might affect myelination, improving neurological conditions. 10 14 36 37 38

Since the studies by Hadders-Algra and collaborators, this subject has also been discussed by other authors in different services. 2 5 14 23 26 27 30 40 41 42 43 Table 2 illustrates the most relevant ones.

**Table 2:** Overview of minor neurological dysfunction findings across studies using the Touwen Neurological Examination 9

Author, year, country	Methodology: sample, assessment, instrument	Results: n (%)
Hadders-Algra, 10 2002, Netherlands	Groningen Cohort; n = 3.162, Touwen Neurological Examination, 6 clusters	Normal exam at neonatal age At 4 y 6 (7%) MND At 9 y 60 (20%) MND At 12y 27 (22%) MND Midly Abnormal exam at neonatal age At 9 y 66 (25%) MND Definitely Abnormal exam at neonatal age At 4 y 9 (12%) MND At 9 y 51 (35%) MND At 12 y 23 (49%) MND
Arnaud et al., 30 2007, France	1.662 preterm with GA < 33 weeks, 245 preterm with GA 33–34 weeks (controls), and 332 full-term controls, assessment at 5 years, Touwen Neurological Examination, 4 clusters	GA < 33 weeks: MND – 591 (47.7%): 515 (41,4%) with simple MND; 38 (3%) with complex MND. 33-34 weeks: MND 61 (31,3%): 60 (30,8%) simple MND; 1 (0,5%) complex MND; Full-Term: MND 65 (22,7%): 63 (22%) simple MND; 2 (0,7%) complex MND
Jongmans et al., 14 1997, United Kingdom	156 preterm with GA < 35 weeks, assessment at 6 years, Touwen Neurological Examination, 10 clusters	48 (31%) with MND: 22 (14%) with simple MND; 26 (17%) with complex MND
Fily et al., 23 2003, France	170 preterm with GA 30 (range: 24–35) weeks and/or birthweight 1,250 g (range: 600–2,690 g), assessment at 5.8 (range: 5–6.5) years, Touwen Neurological Examination, 6 clusters	48 (28%) with MND: 41 (24%) with simple MND; 7 (4%) with complex MND
Peters et al., 13 2011, Netherlands	253 children without mention about gestational age, 167–mainstream education: 8 (range: 5–11), years 86–special education: 9 (range: 5–11), Touwen Neurological Examination, 8 clusters	125 (49%) with MND: 68 (27%) with simple MND; 57 (22%) with complex MND. 30 children with a clinical neurological diagnosis – 25 (83%) with MND: 13 (43%) with simple MND; 12 (40%) with complex MND
Kikkert et al., 21 2013, Netherlands	341 full-term without perinatal risk aged 9 years (range: 8 years and 10 months–9 years and 7 months), Touwen Neurological Examination, 8 clusters	171 (50.1%) with MND: 126 (36.9%) with simple MND; 45 (13.1%) with complex MND
Broström et al., 26 2018. Sweden	80 preterm with GA < 27 weeks and 90 full-term controls without perinatal risk, assessment at a mean age of 6 years 6 months, Touwen Neurological Examination, 4 clusters	Preterm – 29 (36%) with MND: 23 (28.7%) with simple MND; 6 (7.5%) with complex MND. Full-term – 2 (2%) with MND: 2 (2%) with simple MND; 0 with complex MND
Tommiska et al., 4 2020, Finland	64 preterm with GA < 27 weeks, birthweight < 1,000 g, and 30 full-term controls without perinatal risk, birthweight > 2,500 g, assessment at 11.3 (range: 10.6–13.9) years (study group) and 11.8 (range: 70.7–13.8) years (control group), Touwen Neurological Examination, 8 clusters	Preterm – 33 (51.5%) with MND: 25 (39%) with simple MND; 8 (12.5%) with complex MND. Full-term – 7 (23%) with MND; 7 (23%) with simple MND; 0 with complex MND
Erdi-Krausz et al., 5 2021, United Kingdom	27 full-term with risk factor (HIE without CP; study group) and 20 full-term without risk factor (control group), 5–7 years, Touwen Neurological Examination, 4 clusters	6 (22.2%) with MND: 4 (14.8%) with simple MND; 2 (7.4%) with complex MND. Control group: 0 (0%) with MND
Van Hus et al., 41 2014, Netherlands	165 (81 with GA < 30 weeks and/or birthweight < 1,000 g; 84 full-term), Touwen Neurological Examination, 8 clusters	Complex MND: Preterm – 18 (22.2%); Full-term – 2 (2.4%)
Nosko et al., 2 2023, Finland	106 (EPT: *n* = 62; full-term: *n* = 44), assessment between 6.5 and 12 years, Touwen Neurological Examination, 4 clusters	EPT: Simple MND – 26 (42%); Complex MND – 1 (2%). Full-term: Simple MND – 4 (9%)

Abbreviations: CP, cerebral palsy; EPT, extremely preterm; GA, gestational age; HIE, hypoxic-ischemic encephalopathy; MND, minor neurological dysfunction.

Following the GPP study, 10 29 the Étude Épidémiologique sur les Petits Ages Gestationnels (EPIPAGE) project, 27 30 another major population-based cohort, evaluated outcomes in very preterm infants compared with preterm and term infants. Although the results from this cohort were similar to the GPP findings, a higher prevalence of MND was observed at the preschool age, even when compared with GPP children with neonatal neurological abnormalities, further highlighting prematurity as a significant risk factor for MND. Moreover, the proportional reduction in MND among children born before 33 weeks (47.7%), between 33 and 44 weeks (31.7%), and at term (23.3%) highlights the inverse relationship between gestational age (GA) and the prevalence of MND. 27 30

Comparing the frequency of complex MND in preschoolers across the GPP, 10 29 EPIPAGE, 27 30 and the study by Jongmans et al., 14 which focused exclusively on children born preterm, the results suggest that a greater number of clusters considered is associated with a higher reported frequency of this condition: Jongmans et al. (10 clusters) reported a rate of 17% of complex MND, the GPP (6 clusters) reported 12%, and EPIPAGE (4 clusters) reported 3%. This suggests that including clusters such as choreiform dyskinesia and fine manipulative ability may be essential for accurately assessing complex MND in preschoolers. This can also be observed in studies 13 21 4 focusing on school-aged children and pre-adolescents ( Table 2 ).

Among the series described in Table 2 , the highest prevalence of MND was found in a subgroup from the study by Peters et al. 13 (2011), 13 in which 30 children with neurological diagnoses were evaluated, and 25 (83%) of them presented MND during school age. Among the series that assessed children born at term, the highest prevalence of MND was reported by Kikkert et al. 21 (2013), with 171 cases (50.1%) at school age, while among those assessing preterm children, Tommiska et al. 4 (2020) reported the highest prevalence, with 33 cases (51.5%).

### MND x DCD: non-CP motor impairment or motor impairment beyond CP

Conceptually, MND is not a distinct clinical entity but a collection of subtle clinical signs identified through a neurological examination. These signs can occur in both typically-developing and atypical children, and they may co-occur with neurodevelopmental disorders. The condition is associated with motor, behavioral, learning, and cognitive dysfunctions. In terms of motor performance, the impairments observed in MND are often categorized under the broader classification of DCD, an association highlighted in many studies. 10 11 13 26 Thus, MND could be particularly related to the diagnosis of DCD, or non-CP motor impairment, as a motor impairment beyond CP.

Few studies 2 4 5 13 14 44 45 46 have investigated the relationship between MND and DCD using the Movement Assessment Battery for Children (MABC) and the Touwen Neurological Examination; they suggest that most children with complex MND had MABC scores below the cut-off point for DCD, often experiencing motor problems that interfere with daily life. A similar evaluation and conclusions were then provided under the denomination *motor impairment beyond CP* . 5

Peters et al. 13 (2011) evaluated 253 school-aged children without specifying gestational age at birth but included a subgroup attending special education; they identified 30 children with a clinical neurological diagnosis, 6 of whom were in mainstream education. An MABC score below the 5th percentile was associated with complex MND in 54% of the cases, significantly higher than in children with borderline MABC scores (5th–15th percentiles: 17%) and in those with typical MABC scores (below the 15th percentile: 10%) ( *p* < 0.001). Notably, all children with complex MND and MABC scores below the 5th percentile were attending a school for special education. 13

In turn, Jongmans et al. 14 (1997) evaluated 156 school-aged children born preterm, and the occurrence of complex MND was associated with MABC scores below the 5th percentile and between the 5th and 15th percentiles in 46% of the cases for both ranges. Thus, the frequency of DCD was of 92% in those with complex MND, compared with 36% in those with simple MND and 34% in children without MND. 14

It has clinical, diagnosis, and therapeutic implications. Like DCD, according to Hadders-Algra and Touwen, 31 there is sufficient evidence to support that MND represents an increased vulnerability of the brain that might develop into cognitive, emotional, or behavioral problems, as well as a decrease in academic performance. According to the EACD 6 (2019), evidence is emerging that children with DCD often exhibit marked neurodevelopmental immaturities (MND clinical findings), such as choreiform movements of unsupported limbs, mirror movements, as well as signs of impaired ﬁne and gross motor skills. In children with DCD, identifying and classifying MND has treatment and prognosis implications.

Thus, children with motor impairments could benefit from an age-specific, standardized neurological examination, as such an assessment enables the differentiation into two different neurological subtypes: children who have a normal neurological condition or simple MND, and children who have complex MND. Simple MND has limited clinical significance, reflecting the presence of a normal, but non-optimally wired brain, as mentioned before. Complex MND, on the other hand, is more often perinatally acquired and can be considered a distinct and more extensive form of brain dysfunction. The presence of substantial neural dysfunction in children with complex MND is associated with a higher chance of developing motor impairments that interfere with daily life activities, with possible associations with behavioral and cognitive disorders. 2 4 5 13 14 44 45 46

### MND and learning, behavioral, and cognitive impairments

Some studies 1 25 have indicated that motor impairments in early childhood, whether mild or severe, are associated with poorer cognitive and academic outcomes, and lower levels of participation in social leisure activities, hobbies, and sports compared with children without motor impairment.﻿ Major disabilities are usually diagnosed in early childhood, but many minor neurodevelopmental impairments may not be detected before school age, when demands of cognitive and motor skills increase.

This condition has been discussed as early as Dupré (1925), Goldstein (1936), and Strauss and Werner (1943), when they assumed the existence of a syndrome of cerebral dysfunction which manifested in the neurological and behavioral dimensions. 47 During the 1960s and 1970s, many authors suggested the inclusion of the association regarding subtle neurological signs, behavioral disturbances, and learning disabilities under the diagnosis of *minimal brain damage* (1954) and, later, *minimal* (or *minor* ) *brain* (or *cerebral* , or *neurological* ) *dysfunction* (1962). 47

The term *minimal brain dysfunction* , however, was criticized as being too general and heterogeneous. Therefore, very comprehensive signs and symptoms of minimal brain dysfunction were being differentiated into multiple, more specific, and descriptive labels beyond just *hyperactivity* , such as *learning disability* , *dyslexia* and *language disorders* . 48 Nowadays, these and some other diagnoses have been reclassified into a new class called *neurodevelopmental disorders* , a heterogeneous group of conditions that manifest as delay or deviation in the acquisition of expected developmental milestones and behavioral changes. 7

Even though these topics are considered mutually-exclusive diagnoses, they can often be found concomitantly, as behavior and learning disabilities coexisting with either DCD or subtle neurological deficits, associations which have been already largely reported. 7 49 50

Hadders-Algra, Touwen and other authors, 29 31 35 in line with other studies, 51 52 have reported significant relationships involving the presence of MND and motor, learning, and behavioral problems in 6- and 9-year-old children. Most papers 31 53 54 on the subject report on children referred to neurological evaluation either because of learning disabilities or behavioral disorders.

In 1992, Hadders-Algra and Touwen 31 analyzed data from 570 children aged 9 years and concluded that MND is more closely related to learning difficulties (62%) than to behavioral problems (51%). These children belonged to a follow-up study of university-hospital-born children without bias of selection for neither neurobehavioral nor learning problems.

Inversely, publications on the frequency of MND in children showing learning disabilities or behavioral disturbances are still scarce in the literature. Hadders-Algra's team 33 analyzed 104 children who fulfilled the criteria for dyslexia and compared them with a reference group, focusing on the presence of MND. They reported the prevalence of MND in 87% of children with dyslexia, which was significantly higher than that in the reference group of 9-year-olds (21%; *p* < 0.001). Nearly half of the dyslexia group presented with complex MND. The most frequently-described dysfunction was mild fine manipulative disability, found in 80% of the cases, and almost half of the children presented mild dysfunctions in posture and muscle tone regulation, as well as an excess of associated movements.

The cluster type and severity of MND seem to have clinical significance. Fine manipulative disability, problems with coordination, dysfunctional posture, muscle tone and, to a lesser extent, choreiform dyskinesia have been associated with poorer performance in mathematics, reading, and spelling. Associations involving fine manipulative disability and school failure, poorer arithmetic skills, language comprehension, and spelling, as well as associations regarding coordination problems and school failure have been reported. 21

Regarding behavioral problems, the relationship between attention deficit hyperactivity disorder (ADHD) and motor impairment is quite well established. 48 49 55 A group of drug-naive children with ADHD was carefully screened for neurological soft signs assessed through the PANESS, 19 and a strong correlation between ADHD and motor impairment was reported. The findings suggest that neurological soft signs are indicators of the severity of the functional impairment in children with ADHD, as well as the need to include therapeutic measures to aid their motor development. In their opinion, the evaluation of neurological soft signs could even be useful to monitor the effectiveness of the pharmacological treatment 56 . Moreover, they 19 also reported that lower intelligence quotient (IQ) might be related to increased neurological soft signs in children with ADHD.

Previous studies 21 32 35 57 have indicated that the presence of neurological soft signs was associated with lower IQs, but further details were not explored. Little is known about the relationship between MND and IQ. The GPP study 29 enabled the analysis of MND and cognition in healthy 9-year-old children born at term. Kikkert et al. 21 (2013) demonstrated that, in these children, the presence of MND was associated with lower full-scale IQ (FSIQ), performance IQ (PIQ), and verbal IQ (VIQ), in line with previous reports on the subject. 32 35 57 In contrast, no correlation was found between the severity of MND and IQ after adjusting for covariates. Still, two domains of dysfunction – fine manipulative disability and coordination problems – were associated with lower IQ scores; thus, these authors 21 32 35 57 observed prognostic implications related to different MND clusters. Fine manipulative and coordination disabilities were also associated with lower scores on attention, memory and learning and language, whereas other types of MND domains were not. As these studies 21 32 35 57 were performed in children born at term (without risks of developmental disorders), the authors anticipated that these associations would be even stronger in high-risk children. This expectation is based on the higher prevalence of lesions in complex neural circuits, as well as the increased occurrence of MND and cognitive dysfunction commonly observed in this population. 14 21 33 42

### Implications for preventive healthcare and education policy

Prospective effects of perception and motor functions on development and concept formation, as well as on cognitive and behavioral repercussions have been widely discussed and defended by authors such as Kephart, Getman, Frostig, Barsch, Ayres, and Cruickshank. 58 The new motor-development theories state that motor development is embodied, embedded, enculturated, and enabling: it plays an important role for other domains through cascades of neurodevelopment. 59

Nevertheless, our contemporary model of society, based on intellect, prioritizes cognitive performance in lieu of motor performance in educational terms. In practice, it applies to healthy preschoolers, who are submitted to early alphabetization, games and other activities that enhance cognitive performance, instead of employing ludic activities, free play, open-air activities, in contact with nature, which enable proper motor and neurologic maturation. 58

Early screen exposure reinforces this current tendency. The consequences of excessive media exposure, especially before the age of 3 years, include signs of delayed development in terms of language and cognition, as well as socioemotional and behavioral problems. 60 61 Such issues were even more aggravated by the coronavirus disease 2019 (COVID-19) pandemic, which led to the closing of schools and social distancing.

These concepts regarding the relevance of motricity in neurosciences have been revisited in a large cohort of almost 6,000 children aged between 9 and 10 years. 62 The authors found that the effects of regular physical activity on the developing, task-independent (resting-state) functional connectome may play a critical role in the brain's flexibility, response to cognitive demands, and learning. In children, lack of physical activity may adversely affect functional connectivity across brain networks, which can be related to neurodevelopmental and neuropsychiatric disorders. During development, the resting connectome may be significantly benefited by positive factors and enriching experiences, including physical activity. According to their data, 62 physical activity was associated with increased global connectivity and lower ability for further network segregation, both hallmarks of neural maturation. Such arguments endorse the importance of perceptual-motor development, which constitutes the neural maturation basis, and they also point out to professionals the need to recognize subtle neurological abnormalities that might impair the child's cognitive, learning, and behavioral development. 62

Thus, we believe that calling attention to early subtle motor impairments may have implications for preventive healthcare and education policies, especially for toddlers and preschoolers.

Regarding an established neurodevelopmental disorder such as DCD, the most robust evidence is currently focused on task-oriented approaches. This modality is centered on the person, on the objective, task, and context, targeting functionality rather than normality, with active involvement of caregivers. The best examples are neuromotor task training (NTT) and the cognitive orientation to daily occupational performance approach (CO-OP). The process-oriented approach has fewer evidence of great results. 6

In preschool children, however, it is not yet possible to establish a diagnosis of DCD, but it is now possible to identify MND. Considering the relevant associations involving the persistence of this set of subtle neurological signs and future motor performance problems, such as DCD, in addition to other cognitive and behavioral impairments, early stimulation of motor skills is even more necessary than already recommended for typical children. Stimulating the development of psychomotor skills can only be performed more effectively through extensive experimentation, in a conducive environment, through trial and error, which is made possible through activities authentically incorporated into everyday life, continually challenging, and highly-focused on social engagement. 63

Minor neurological dysfunction remains a subject of debate in the scientific literature. Some critics argue that the statistically significant associations between MND findings and learning difficulties lack meaningful clinical relevance. Others 12 suggest that MND may be a transient phenomenon that resolves over time or merely reflects a temporary delay in neurodevelopmental maturation.

On the other hand, Hadders-Algra and Touwen 31 (1992) argue that it is unlikely that the signs of minor dysfunctions identified in neurological examinations reflect typical central nervous system (CNS) function, asserting that such deviations should not be considered part of a child's normal neurological repertoire. This perspective is particularly relevant in cases of complex MND, which is associated with specific etiological factors, distinct pathophysiological mechanisms, and well-documented cognitive and behavioral impacts. A practical application of this concept can be observed in the context of premature children and those with low birth weight, as evidenced in Table 2 , which also highlights several studies demonstrating how the concept of MND can be effectively incorporated into the pediatric neurology practice. However, for the establishment of MND, it is essential that signs of dysfunction appear as a functionally-related cluster; a single isolated finding is insufficient. This criterion is crucial to prevent the overestimation of subtle neurological signs, which could lead to an unnecessary overdiagnosis of motor dysfunction.

The ideal approach to MND should balance its recognition as a relevant diagnostic tool with caution against overinterpreting isolated findings. Such balance can help refine both the clinical practice and research, ensuring that MND is applied in a methodical and scientifically grounded manner. 13 14

### Limitations

Although the narrative review design was chosen to provide an overall summary with interpretation and critique, we acknowledge its methodological limitations. Our intention at this stage is indeed to provide general background, following a reasoning process grounded in the literature, from historical aspects to MND associations with neurodevelopmental disorders. To date, no comprehensive review on this topic has been conducted. However, a stronger methodological review may be necessary, which could be achieved using the related series.

### COULD MND BE A TYPE OF NON-CP MOTOR IMPAIRMENT?

Currently considered an entity, a group of clinical motor signs, MND, especially complex MND, seems to be closely related to DCD, which is considered a non-CP motor impairment. Thus, from our point of view, the answer to this question may be: Yes, it could, as evidenced by several studies.

That said, DCD and MND should get more emphasis on pediatric and neurological evaluations, besides cognitive, behavioral, and learning abilities. Children with motor impairment or neurodevelopmental findings could benefit from an age-specific, standardized neurological examination, such as the Touwen Neurological Examination; such an assessment enables the differentiation into children who have a normal neurological condition, with limited clinical significance, such as simple MND, and children who have complex MND, which can be considered a more extensive form of brain dysfunction, with a higher chance of developing motor problems that interfere with daily life activities (DCD inclusive), as well as the development of learning, cognitive or behavioral disorders, which, all in all, means that these children particularly deserve special attention regarding social, educational and health care resources.

Furthermore, with the aim of supporting neural maturation and cognitive health, motor activity seems to play an important role, more than what is already recognized for the physical, mental, and neurological health of typical children, based on neurosciences data. By discussing these ideas, even beyond neurological dysfunctions, different approaches to education could have a positive impact on neurological development.

While the concept of MND is still under debate, its careful application as a diagnostic tool offers valuable insights into neurodevelopmental challenges, particularly in high-risk populations. By adhering to rigorous diagnostic criteria and maintaining a balanced perspective, MND can continue to evolve as a meaningful framework in the pediatric neurology practice and research.
